# Adverse genomic alterations and stemness features are induced by field cancerization in the microenvironment of hepatocellular carcinomas

**DOI:** 10.18632/oncotarget.16231

**Published:** 2017-03-15

**Authors:** Darko Castven, Michael Fischer, Diana Becker, Stefan Heinrich, Jesper B. Andersen, Dennis Strand, Martin F. Sprinzl, Susanne Strand, Carolin Czauderna, Stefanie Heilmann-Heimbach, Stephanie Roessler, Arndt Weinmann, Marcus A. Wörns, Snorri S. Thorgeirsson, Peter R. Galle, Matthias S. Matter, Hauke Lang, Jens U. Marquardt

**Affiliations:** ^1^ Department of Medicine, Johannes Gutenberg University, Mainz, Germany; ^2^ Department of Surgery, Johannes Gutenberg University, Mainz, Germany; ^3^ Department of Health and Medical Science, Biotech Research and Innovation Centre, University of Copenhagen, Copenhagen, Denmark; ^4^ Department of Genomics, Institute of Human Genetics, Life & Brain Center, University of Bonn, Bonn, Germany; ^5^ Institute of Pathology, University Hospital Heidelberg, Heidelberg, Germany; ^6^ Laboratory of Experimental Carcinogenesis, Center for Cancer Research, National Cancer Institute, NIH, Bethesda, MD, USA; ^7^ Department of Pathology, University of Basel, Basel, Switzerland

**Keywords:** liver cancer, microenvironment, hepatocarcinogenesis, stemness features, field effect

## Abstract

Hepatocellular Carcinoma (HCC) commonly develops in chronically damaged liver tissues. The resulting regenerative and inflammatory processes create an adverse milieu that promotes tumor-initiation and progression. A better understanding of the hepatic tumor-microenvironment interaction might infer profound therapeutic implications.

Integrative whole genome and transcriptome analyses of different tumor regions, the invasive tumor border and tumor-surrounding liver (SL) were performed to identify associated molecular alterations and integrated with our existing HCC database. Expression levels and localization of established CSC markers were assessed in pre-neoplastic lesions and confirmed in two independent patient cohorts using qRT-PCR, immunohistochemistry and immunofluorescence.

Our results indicate that genomic and transcriptomic profiles between SL and different tumor regions are quite distinct. Progressive increase in genetic alterations and activation of pathways related to proliferation as well as apoptosis were observed in the tumor tissue, while activation of stemness markers was present in cirrhotic SL and continuously decreased from pre-neoplastic lesions to HCC. Interestingly, the invasive tumor border was characterized by inflammatory and EMT-related gene sets as well as activation of pro-survival signaling. Consistently, integration of gene expression signatures with two independent HCC databases containing 300 HCCs revealed that border signatures are predictive of HCC patient survival.

Prognostic significance of the permissive liver microenvironment might be a consequence of a pro-oncogenic field effect that is caused by chronic regenerative processes. Activation of key oncogenic features and immune-response signaling indicates that the cross-talk between tumor and microenvironment might be a promising therapeutic and/or preventive target.

## INTRODUCTION

Hepatocellular carcinoma (HCC) ranks among the most common cancers worldwide [1]. In the vast majority of patients HCC develops on the basis of an underlying chronic liver disease, whereby the chronic liver damage induces subsequent regenerative and inflammatory processes [2]. A constant remodeling of the diseased liver parenchyma and activation of immune-cell mediated inflammation creates an adverse milieu that promotes HCC development [3]. As a result, a significant phenotypic and molecular heterogeneity is observed in HCC that hampers therapeutic progress and, to date, sorafenib remains the only approved therapy for advanced HCC [4].

It is well recognized that acquisition of pre-neoplastic (epi-)genetic alterations in the hepatic microenvironment induces a continuum of morphologic changes from chronic inflammatory cell death over cirrhosis to dysplastic lesions which promotes malignant transformation [5]. Intense cross-talk between cancer cells and stromal/immune cells further promotes HCC development and progression in the majority of HCCs [6]. Also, activation of pro-inflammatory cytokines (e.g. acquisition of autocrine IL6 signaling in hepatic progenitor cells) induces a broad range of effects on a variety of resident and non-resident cells (e.g. immune cells) and can be considered a key oncogenic driver in HCC development [7]. Consistently, we have demonstrated that activation of immune-related signaling pathways in dysplastic lesions and early HCC is important for the sequential evolution of liver cancer and precedes the acquisition of malignant features in progressed HCC [8]. Therefore, activation of molecular changes involved in inflammation as well as cancer should be considered in the pursue to identify novel therapeutic targets [3]. Importantly, activation of inflammation related gene-sets not only impairs the development of HCC, but possesses profound prognostic implications [9]. A recent study further showed that gene expression signatures generated from surrounding non-tumor liver tissue could accurately predict the patient outcome in HCC, whereas signals from the tumor did not provide a meaningful clinical association [10].

Several immune response-related and pro-oncogenic molecules induce opposing effects when activated in diverse parenchymal and non-parenchymal cell types (e.g. immune cells versus hepatocytes) and during different states of the chronic liver disease (e.g. inflammation, fibrosis, cirrhosis) which underlines the critical importance of the interaction of signals from the microenvironment and the tumor cells for tumor initiation and progression [11, 12]. Other aspects of the tumor-microenvironment cross talk are synergistic and amplify the malignant potential of the tumors [6]. Therapeutically, different cell types might augment the anti-cancer activity of sorafenib. Further, several studies clearly showed that pro-oncogenic changes in the hepatic microenvironment during chronic liver inflammation are orchestrated by the interaction of parenchymal cells with diverse types of non-parenchymal cells [5, 13]. Therefore, a better understanding of the tumor-microenvironment interaction might open therapeutic options [14]. In the here presented study, we analyzed the genome and transcriptome profiles of tumor tissue, the invasive tumor margin and peritumoral liver tissue of HCC patients from a Western cohort. Our results demonstrate that the hepatic microenvironment is critical for malignant progression of HCC. While genetic alterations continuously increased from the peritumoral tissue to the tumor core, prognostic adverse transcriptomic signals and stemness features were activated in the invasive tumor margin of the tumor surrounding liver tissue. These observations suggest that the chronic inflammation creates a pro-oncogenic field effect and should be considered a hallmark of liver cancer.

## RESULTS

### Characterization of the molecular profiles in different tumor regions

**Figure 1:**
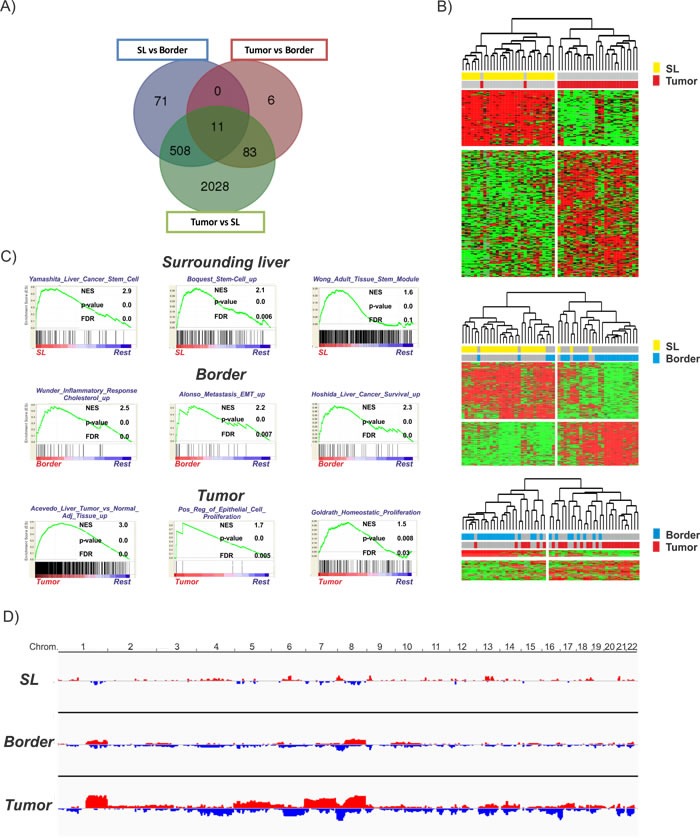
Transcriptomic and genomic profiles of the different regions **A.** Venn diagram demonstrating the overlap between the different gene expression signatures. **B.** Unsupervised hierarchical cluster analysis of the different regions based on the corresponding significant genes (SL vs tumor (2630 genes): upper panel; SL vs border (590 genes): middle panel; border vs tumor (100 genes): lower panel) **C.** Gene set enrichment analysis (GSEA) for each of the regions in comparison to all other regions (rest). Normalized enrichment score (NES) reflects degree of overrepresentation for each group at the peak of the entire set. Statistical significance calculated by nominal P value of the ES by using an empirical phenotype-based permutation test. **D.** Graphical representation of the genetic alterations in each region determined by DNAcopy. Amplifications are depicted and red and losses are depicted in blue.

The importance of the chronic inflammatory liver diseases for HCC initiation and progression has been repeatedly demonstrated [5]. To characterize in more detail the cross-talk between tumor cells and the diseased microenvironment and define key molecular mechanisms leading to cancer progression, we macroscopically dissected non-neoplastic tumor-surrounding liver tissue (SL) from the invasive tumor margin/ border (B) as well as the core tumor tissue (T). First, we assessed the global transcriptome profiles for each of the regions. As expected, the highest number of differentially expressed genes was found between T and SL (2630 genes), followed by B vs SL (590 genes) (Supplementary Table S3). Transcriptomic profiles of T and B showed the highest similarity and only 100 genes showed significant differences (Figure 1A). The identified gene expression signatures significantly separated the different regions, thus validating that the genes effectively stratify for each region (Figure 1B). Not unexpectedly, subsequent pathway analysis using GeneGo and IPA showed enrichment of genes related to pro-proliferative signaling and DNA damage response (CCND1, CD4, ABCC4, APEX1, MAPK9, BMI1, MAT1A, MLH1, FADD, FOS, PIK3R1, FOS, RAD51C, XRCC3) for the T vs SL as well as B vs SL signatures (Supplementary Table S4). Furthermore, both the T vs B and B vs SL signature showed activation of functional networks related to immune response, inflammation and IL-6 signaling (CXCL12, CUL1, CDK5, CD82, PIK3R1, SMARCA4, ESR1, IKBKG, SPP1, IL17RA, EGR1, IGF1, IGFBP1, IFNAR1), confirming the crucial role of chronic inflammation and related molecular pathways for hepatocarcinogenesis [7]. Additionally, molecules related to adverse and pro-metastatic features were also highly activated/repressed in the invasive tumor margin (CDH1, ABCG5, APOA1, ID2, LAMC1, CTNNA1, MGMT, HNF4A, ALDH8A). Next, gene set enrichment analysis (GSEA) was performed to delineate the activated gene sets characteristic for each region. GSEA confirmed the activation of genes involved in pro-oncogenic signaling and proliferation in the T region (Figure 1C; lower panel) as well as inflammation in the B region (middle panel). Further, enrichment of gene sets commonly associated with EMT, metastatic traits as well as survival was observed for the B region, indicating a potential association to the outcome of HCC patients. Interestingly, GSEA revealed an abundance of gene sets associated with (cancer-) stemness in SL regions (Figure 1C; upper panel). These results indicate that the tumor margin (B) is enriched in inflammatory gene sets and shows activation of adverse signaling pathways, while tumor tissues mainly show activation of proliferative genes. Further, our results indicate that the hepatic microenvironment contributes to a stemness phenotype observed in many human HCCs.

We next assessed the somatic genetic alterations present in the different regions by profiling the corresponding tissues using Illumina OmniExpress arrays followed by GISTIC 2.0 analyses. Overall, progression from SL to T showed a continuous increase in genetic alterations (Figure 1D; Supplementary Figure S2). As expected, recurrent changes of B and T regions involved gains in 1q and 8q as well as copy number losses in 8p (Figure 1D) [15]. GISTIC analyses based on corresponding SL further identified 15 and 8 significantly reoccurring focal amplifications in T and B regions, respectively (Supplementary Table S3). Notably, the only commonly amplified regions in T and B involved 7q11.21 while other well characterized driver oncogenes such as hTERT (5p15.33) were only observed in tumors. Among 30 deletion events in T and 39 in B regions, PTX4 (16p13.3), KIF20B (10q23.31), SYCP2 (20q13.33) and 9p21.3 (CDKN2A and CDKN2B, MTAP) were prominent in both T and B. These results confirm that alterations of the genome play a central role for tumor cell proliferation in T and B. Further, transcriptome changes observed in SL might be predominantly driven by other mechanisms (e.g. epigenetics) and potentially induced by the chronic inflammation as well as the tumor-microenvironment cross-talk.

### Activation of pro-proliferative gene sets in the tumors

**Figure 2:**
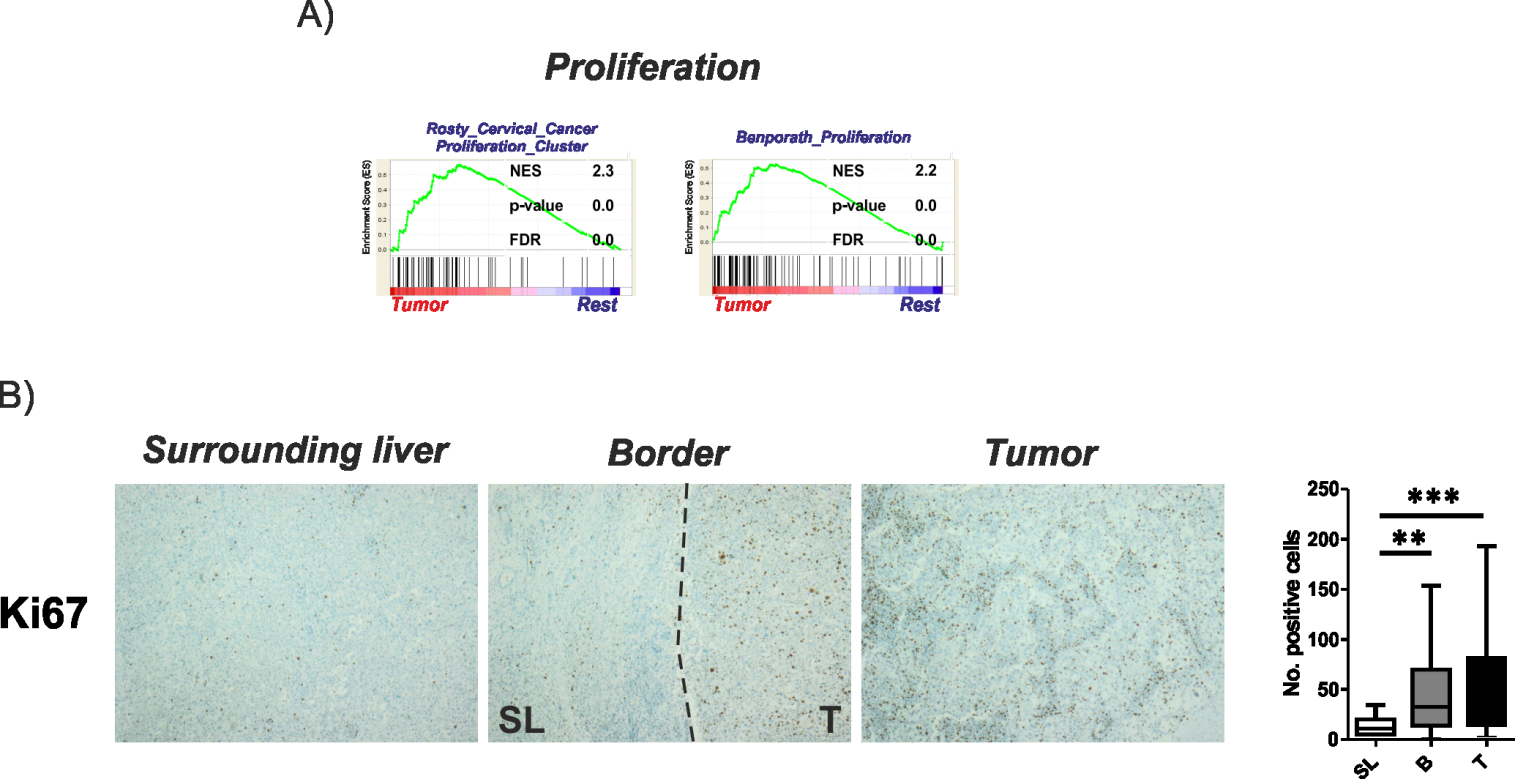
Activation of proliferation in tumor tissues **A.** Gene set enrichment analysis (GSEA) of the tumor regions in comparison to all other regions (rest) indicate an activation of proliferative gene sets. Normalized enrichment score (NES) reflects degree of overrepresentation for each group at the peak of the entire set. Statistical significance calculated by nominal P value of the ES by using an empirical phenotype-based permutation test. **B.** Proliferation of cells determined by Ki67 staining. Dashed bars indicating the separation between SL and T regions. Right panel shows the graphical representation as number of positive cells estimated based on 10 randomly selected view fields (20x magnification). Statistical evaluation based on Friedman- test for multiple group comparisons followed by Dunns posthoc test. (*n* = 22; P-values: *≤ 0.05; **≤ 0.05; ***≤ 0.001). The data are presented as mean fold differences ± SD.

Our molecular analyses indicated a predominant activation of proliferative signaling in the T region that might reflect tumor cell proliferation thereby potentially supporting tumor expansion. Extension of the GSEA for the T region confirmed this finding and demonstrated an enrichment of gene sets associated with deregulation of cellular genes particularly related to tumor cell proliferation in cervical cancer [16] as well as genes generally associated with proliferation [17] in this region (Figure 2A). Notably, despite the proliferative potential of these genes and frequent activation in cancer, no association to the outcome of patients could be established suggesting that the high proliferation observed in many solid tumors might not necessarily confer to prognostic traits [17]. To confirm that the activation of proliferation genes indeed confers to proliferation in the tumors, specimens were stained for Ki67 expression (Figure 2B). As expected, significantly higher Ki67 levels were observed in T and B in comparison to SL. Notably, although proliferation of cells in T was generally highest, expression levels did not significantly differ to those observed in the B regions (Figure 2B).

### Activation of stemness in the peritumoral tissue

**Figure 3:**
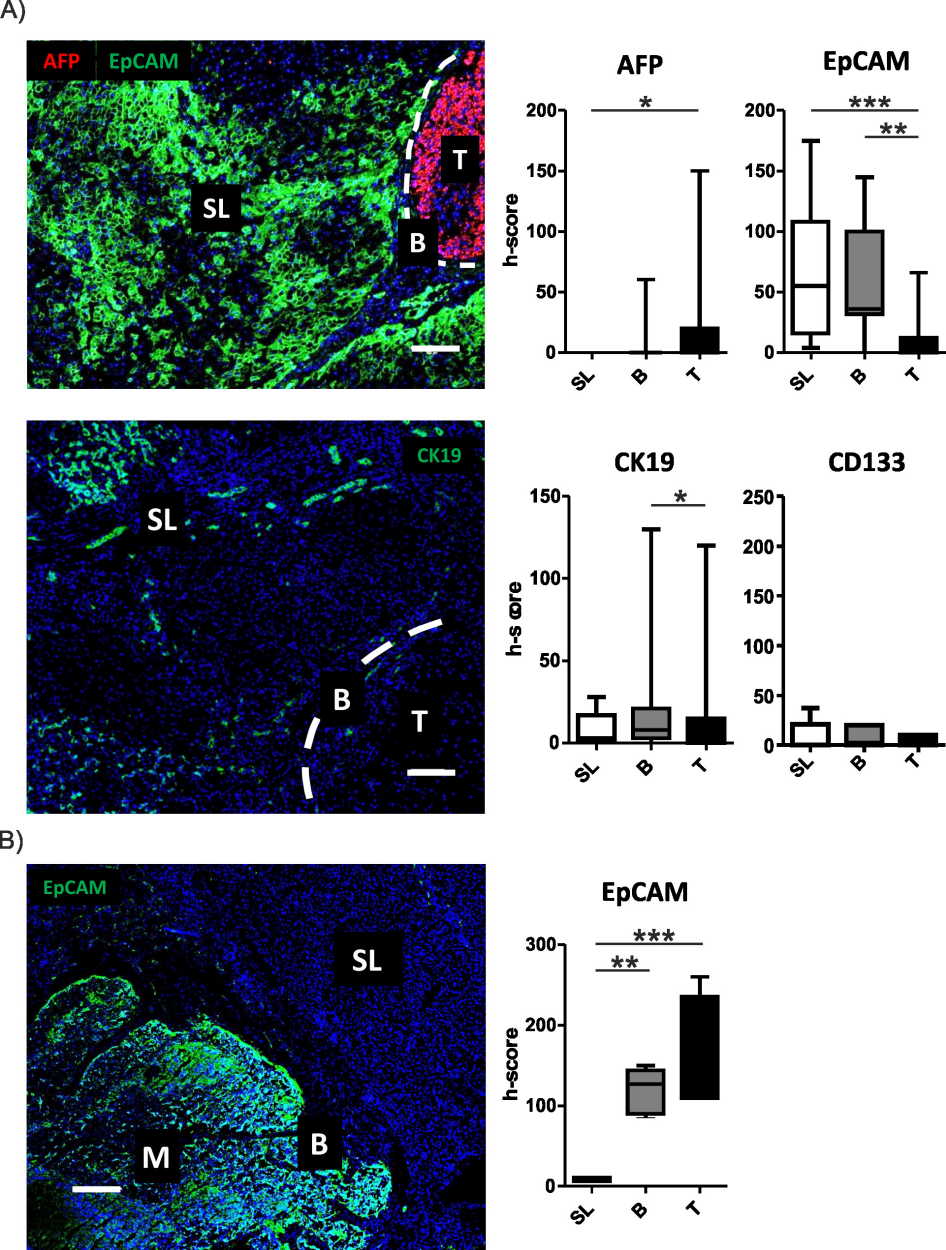
Activation of stemness markers in peritumoral tissues **A.** Activation of stemness marker in the different regions was determined by confocal imaging. Representative images for AFP (red) and EpCAM (green) staining (upper panel) and CK19 (lower panel) containing all different regions (T = tumor, B = border, SL = surrounding liver) are displayed. Dashed bars indicating the separation between SL and T regions. White bar representing 100μm. Graphical representation and statistical evaluation (right graphs) for each marker based on h-score and Friedman- test for multiple group comparisons followed by Dunns posthoc test. (n = 15; P-values: *≤ 0.05; **≤ 0.05; ***≤ 0.001). The data are presented as mean fold differences ± SD. **B.** Activation of stemness marker EpCAM (green) by confocal microscopy. Images contains representation of all different regions (M = metastasis, B = border, SL = surrounding liver). White bar representing 200μm. Graphical representation and statistical evaluation (right graphs) for each marker based on h-score and Friedman- test for multiple group comparisons followed by Dunns posthoc test. (*n* = 5; P-values: *≤ 0.05; **≤ 0.05; ***≤ 0.001). The data are presented as mean fold differences ± SD.

Since our molecular analyses indicated an activation of stemness in the SL region, we next assessed the gene expression levels of specific HCC/differentiation markers (AFP, GPC3, albumin) as well as the selected (cancer-) stemness markers (EpCAM, CD133, CK19) and pluripotency genes (NANOG). As expected, a strong activation in expression levels of AFP and GPC3 as well as a concomitant downregulation of albumin levels were observed from SL to T (Figure 3A; Supplementary Figure S3). Notably, expression of AFP was generally low and undetectable by qRT-PCR in the majority of cases. Consistently, levels of the stemness and pluripotency markers showed a significant decrease from SL to T (Figure 3A; Supplementary Figure S3). Of note, expression of well-known progenitor cell marker CK19 was highest in peritumoral tissue and almost absent in tumor tissue [18]. However, two of the investigated tumors showed a high positivity leading to a high variability and explaining the missing statistical significance between SL and T (Figure 3A). Results for AFP, GPC3 and EpCAM could further be validated in an independent validation cohort of HCC patients as well as cirrhotic livers without HCCs (Supplementary Figure S4, Table 1B). Importantly, activation of EpCAM did not show significant differences in cirrhotic livers in the absence of HCC, confirming that induction of EpCAM positive cells in the tumor-surrounding liver is predominantly seen in the context of hepatocarcinogenesis (Supplementary Figure S4). Interestingly, similar results could also be revealed during sequential evolution of liver cancer (Supplementary Figure S5). RNA sequencing in patients with synchronous co-existence of pre-neoplastic lesions as well as HCC confirmed a continuous decrease in stemness genes from SL over dysplastic lesions to progressed HCC.

To confirm that the diseased hepatic tumor microenvironment is the critical determinant of stemness activation, we next assessed expression levels of the stemness genes in livers containing metastasis from different primaries (n = 5). Importantly, we found that these markers were not induced in the non-diseased liver tissue (i.e. in the absence of chronic liver damage) (Figure 3B, Supplementary Figure S6). However, consistent with an aggressive phenotype of metastases, high levels of the markers were detected in metastatic cells (Figure 3B; Supplementary Figure S6). Together, these results corroborate that the hepatic microenvironment plays a crucial importance for the acquisition of stemness traits in HCC.

### Activation of immune cells in the invasive tumor border

**Figure 4:**
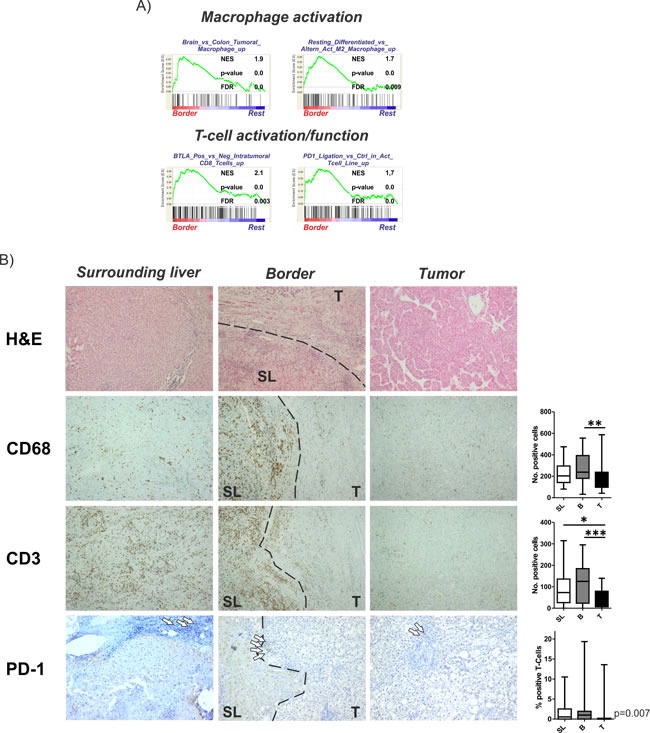
Activation of inflammatory gene sets and immune cells in the invasive tumor margin **A.** Gene set enrichment analysis (GSEA) of the surrounding liver regions in comparison to all other regions (rest) indicate an activation of gene sets involved in macrophage as well as T cell activation/function. Normalized enrichment score (NES) reflects degree of overrepresentation for each group at the peak of the entire set. Statistical significance calculated by nominal P value of the ES by using an empirical phenotype-based permutation test. **B.** Representative H&E stainings are shown in the upper graph. Immunohistochemistry of CD68 and CD3 demonstrating activated macrophages and T cells. Lower panel shows representative images of PD-1 staining reflecting impaired T cell function. Dashed bars indicating the separation between SL and T regions. White arrows indicating selected positive cells. Right panels show the corresponding graphical representations as number of positive cells estimated based on 10 randomly selected view fields (20x magnification). Statistical evaluation based on Friedman- test for multiple group comparisons followed by Dunns posthoc test. (*n* = 22; P-values: *≤ 0.05; **≤ 0.05; ***≤ 0.001).

To confirm the activation of inflammatory gene sets and dissect the corresponding immune cells reflected by the molecular changes, we limited our GSEA query to gene sets with association to immune-related properties. We observed a significant enrichment of gene sets related to alternative M2 macrophage activation that might exert pro-tumorigenic function (Figure 4A). Furthermore, molecular signals resembling non-hypoxic macrophages that display impaired anti-tumor response were enriched in B regions which might further decrease the immune response and promote immune escape of HCCs. Consistently, a significant increase of CD68 could be demonstrated in B vs T regions (Figure 4B; upper panel). Additionally, gene sets associated with activated tumor-infiltrating CD8 T cells were enriched in the B regions. However, we also recognized activation of genes indicative of PD-1 function commonly associated with exhaustion anti-tumoral T cell function and properties, potentially resulting in impaired immune-surveillance. Consistently, we observed a significantly higher number of CD3-T cells as well as increased PD-1 expression in the SL and B region compared to T (Figure 4B). Together, these results highlight the importance of immune-related mechanisms in the invasive B region for tumor progression.

### The invasive tumor border is important for HCC patient outcome

**Figure 5:**
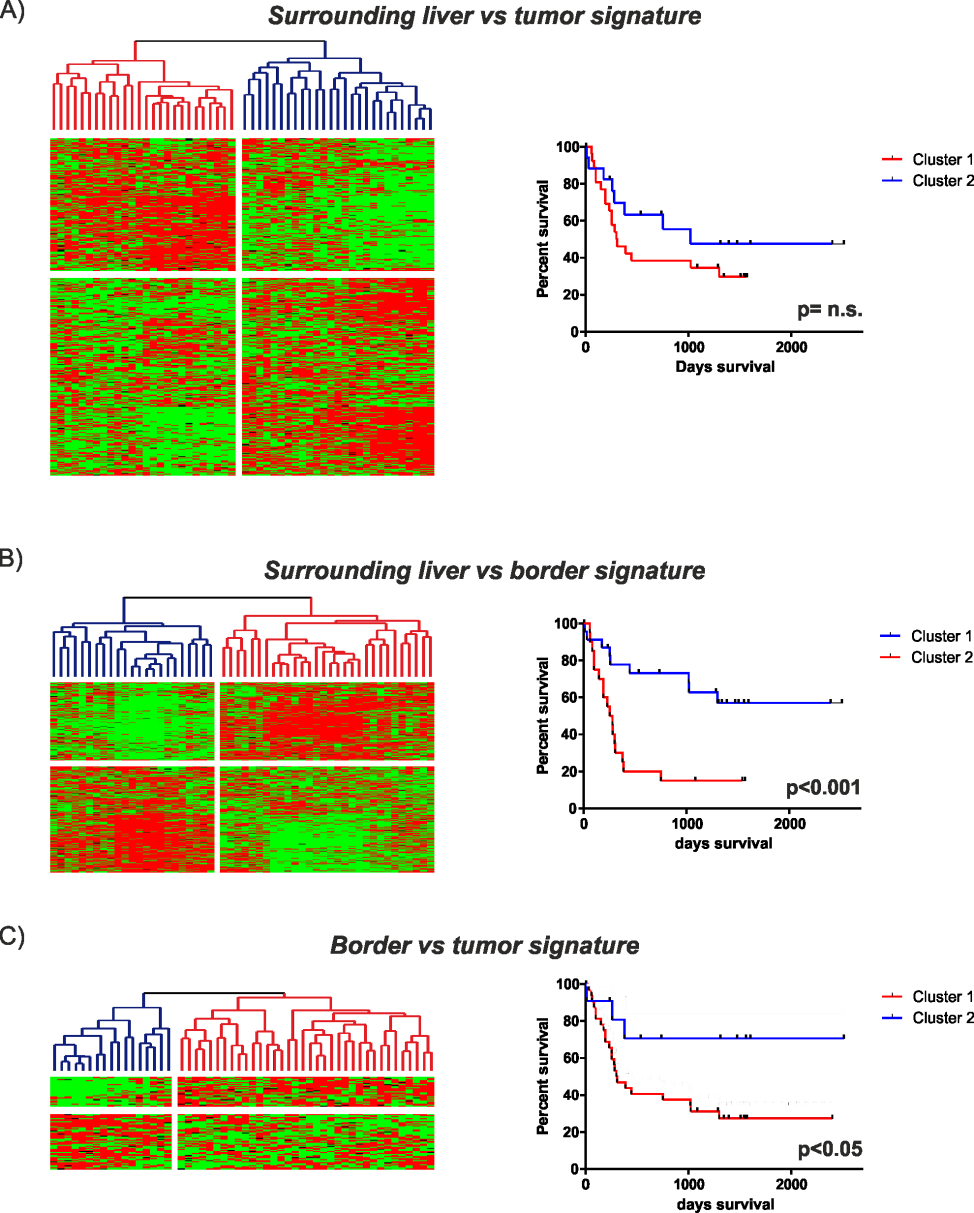
Prognostic implications of the identified gene expression signatures Integration of the different gene expression signatures with our previously published dataset of 53 HCC patients [19]. Unsupervised cluster analyses (left) and Kaplan-Meier plots of overall survival (right) for each of the identified signatures are shown: **A.** SL vs tumor (2630 genes): upper panel; **B.** SL vs border (590 genes): middle panel; **C.** border vs tumor (100 genes): lower panel).

Finally, we tested the clinical significance of our identified SL, B and T signatures and integrated all three signatures with our previously published gene expression dataset of 53 human HCC. [19] Subsequent Kaplan-Meier analysis showed that, despite the high proliferative activity, the T vs SL signature did not possess prognostic significance (Figure 5A). However, both the B vs SL and B vs T signatures independently classified HCC patients according to survival (Figure 5B and 5C). The prognostic impact of both signatures could further be confirmed in an independent cohort from 247 HCC patients (Supplementary Figure S7). Overall, the prognostic impact of signatures derived from signals of the B region underlines the clinical importance of the invasive tumor-front that might be helpful to identify novel therapeutic targets.

## DISCUSSION

The notion that HCC patients display two diseases that are inextricably linked to each other, i.e. a chronic liver disease and a malignant tumor, is increasingly recognized, whereby the diseased hepatic microenvironment significantly promotes cancer initiation and progression while concomitantly limiting aggressive therapeutic approaches [20]. Therefore, the importance of the chronic inflammatory liver disease can be considered a hallmark feature of HCC and has become focus of intense research [3, 21]. We here provide evidence that chronic changes of the liver microenvironment induce an adverse pro-oncogenic niche that might lead to the activation of stemness features and pre-dispose to liver cancer development. Heterogeneous patterns of molecular alterations present in tumor surrounding liver tissue suggest that a potential field cancerization might significantly contribute to hepatocarcinogenesis [22]. Further, our transcriptome analyses indicate that the cross-talk between the inflammatory microenvironment and the tumor cells in the invasive tumor border might be an important determinant of the patient prognosis while tumor cell growth is mainly driven by proliferative signaling induced in the tumors.

To address the importance of distinct peritumoral and tumoral regions for hepatocarcinogenesis we analyzed the molecular profiles of tumor-surrounding liver tissue, the invasive tumor border and core tumor tissue by genome-wide approaches and at different molecular levels. The transcriptome analyses confirmed a distinct gene expression profile for each of the different regions (Figure 1). As expected, most abundant differences were observed between T and SL regions and mainly centered around functional networks involved in tumor cell proliferation as a key feature of tumor expansion [23]. Consistently, the tumor region showed the highest amount of cycling cells (Figure 2B). Concomitantly, most significant genetic alterations were also observed in T regions and involved known driver genes of hepatocarcinogenesis such as hTERT and CDKN2A and CDKN2B [24, 25]. An interesting observation is the activation of stemness gene sets and pathways as well as established (cancer-) stemness markers in the tumor surrounding liver tissue as well as the invasive B region (Figure 3; Supplementary Figure S3) [26, 27]. The notion that the acquisition of stemness is an important characteristic of malignant transformation is well recognized and expansion of cells that display progenitor cell features is frequently observed in the majority of chronic liver diseases [2, 28, 29]. A recent study further utilized a monoclonal antibody against 1B50-1 to detect a population of cells with CSC properties in human HCCs [30]. In concordance with our findings, these CSCs were located in the surgical margins of primary HCC and possessed prognostic as well as therapeutic implications. Our results, therefore, support the hypothesis that the permissive hepatic microenvironment induces a pro-oncogenic field effect which activates stemness traits and ultimately promotes tumor development and progression [22, 31, 32]. The relatively low number of genetic alterations such as copy-number changes further suggests that gene expression changes in the SL region are mainly driven by other mechanisms, e.g. epigenetic mechanisms, which might support the concept of an epigenetic progenitor cell origin in HCC that is induced by the chronic inflammatory liver disease [33]. Notably, whether the cellular origin of the cells resembles hepatocytes or stem progenitor cells remains uncertain [7, 34, 35]. Although the decrease in stemness markers during sequential hepatocarcinogenesis from SL over dysplastic lesions to established HCCs (Supplementary Figure S5) favors the hypothesis that these cells contribute to tumor development, invasion of stem-like cancer cells from the tumor as a reflection of malignant progression cannot be excluded [36, 37]. Our analyses also suggest that, while the proliferative properties were predominantly acquired by tumor cells in the T region, B and SL show additional activation of immune-related and pro-metastatic signaling. Activation of inflammatory gene sets in tumor-surrounding liver tissue is a hallmark feature of HCC and associated with a poor clinical outcome [10]. Consistently, a significantly higher number of both macrophages and T cells was seen in SL and B regions compared to T regions (Figure 4). Despite several reports that show differences in the (immune-)cellular composition in the B and SL regions, a recent study also confirmed a potential therapeutic impact of this region [38]. Treatment with sorafenib induced significant changes to the HCC microenvironment by affecting macrophage polarization and inhibited accumulation of adverse M2 polarized tumor-associated macrophages at the tumor margin or within the peritumoral area. We also observed an increased number of CD3 T cells in the SL and B region, a feature that is commonly associated with an increased tumor-cell clearance and favorable outcome [39]. However, the dominant activation of T cells with enrichment for PD-1 function suggest that these cells might display impaired antitumor activity thereby leading to evasion of tumor-immuno-surveillance and poor outcome [40–42]. Notably, activation of check-point genes such as PD-1/PDL-1 and CTLA4 could be linked to a poor clinical outcome in HCC [40, 41]. Therefore, results of our study owes to the promise of immunotherapeutic approaches for liver cancer [43]. Importantly, the design of our study does not allow the prediction which of the individual patients will show a favorable or adverse outcome. Therefore, the activation of adverse signaling pathways and putative prognostic implications within the B regions are strictly associative. Since several reports suggest that infiltrating CD8 T-cells in the tumor border might also confer to a favorable outcome [39], these observations clearly require a detailed functional validation as well as individual confirmative analyses. Nevertheless, our results demonstrate a therapeutic and prognostic importance of the tumor/liver border which is not assessed in the current clinical routine (e.g. histology). Our results, therefore, suggest that novel diagnostic strategies should include the molecular and histological evaluation of the invasive tumor border. In line with this, integration of our transcriptome signatures with two independent cohorts of HCC patients clearly confirmed that the activation of inflammatory and adverse signaling pathways in the B regions has a strong association with the outcome of HCC patients (Figure 5; Supplementary Figure S7) [15, 19]. Together, the here presented results provide a detailed molecular and phenotypic characterization of the different peritumoral, border as well tumor regions and highlight the critical importance of the diseased hepatic microenvironment for HCC initiation and progression. The indicated prognostic significance of the cross-talk between tumor cells and microenvironment further underlines the recent success of immune check-point inhibitors in HCC and makes HCC a prime target for immunotherapeutic interventions [43].

## MATERIALS AND METHODS

### Patient data and nucleic acid extraction

Tissue from 28 patients with confirmed HCC undergoing resection at the Department of Surgery, University of Mainz, Germany were collected following patient informed consent and local ethics committee approval. Validation cohort of 20 patients was obtained from the Institute of Pathology, University of Basel, Switzerland. Clinicopatholigical details are provided in Supplementary Table S1. Tissue was macroscopically dissected into tumor tissue (T), tumor margin/ border (B) (approximately 2-4mm from each region) and peri-tumoral tissue (SL) (Supplementary Figure S1). Total RNA was extracted using the Qiagen RNEasy mini Kit (Qiagen GMBH, Hilden, Germany) following the manufacturer's instructions. RNA quantity and purity were estimated using a Nanodrop ND-1000 Spectrophotometer (NanoDrop Technologies, Wilmington, DE), and integrity was assessed by Agilent 2100 Bioanalyzer (Agilent, Palo Alto, CA). DNA was extracted using Qiagen Qiamp DNA Kit (Qiagen GMBH, Hilden, Germany) following the manufacturer's instructions.

### Gene expression analysis

A total of 200 ng RNA was linearly amplified as recommended by the manufacturer (Ambion, Austin, TX) and analyses were performed as described before [44]. The microarray datasets have been deposited to Gene Expression Omnibus database (http://www.ncbi.nlm.nih.gov/geo, accession number: GSE84598). Details of the analyses are provided in the Supplementary materials and methods.

### Immunohistochemistry and confocal microscopy

Diagnosis of HCC was established by expert pathologists. Tissue was either fixed in 4% formaldehyde and embedded in paraffin or preserved for cryosections and cut in 3-5 μm sections. Antibodies and conditions for fluorescence and immunohistochemical staining are listed in Supplementary Table S2. Fluorescently stained tissues were viewed by a Zeiss LSM 710 NLO confocal microscope with 25x magnification objective and Tile Scan function. Quantification was performed using H-score. H-score was determined based on the intensity of the staining (0-3) and percent of the positive cells (0-100%) in a fixed view field. H-score was calculated using the next formula: H-score = 1x(% cells with score 1)+2x(% cells with score 2)+3x(% cells with score 3). Immunohistochemistry was performed by automated immunostaining with iVIEW DAB detection kit (Ventana Medical System, Roche, Mannheim, Germany) according to the company's protocols. Monoclonal anti-CD68 (dilution 1:70, DakoCytomation), Ki67 antibodies (dilution 1:200, Rockland) and anti-CD3 antibodies (dilution 1:100, Santa Cruz) were used. Anti-EpCAM antibodies (dilution 1:80, Cell Signaling), anti-GPC3 antibodies (dilution as given by provider, Ventana Medical System, Roche, Basel, Switzerland), anti-AFP antibodies (dilution 1:400, DakoCytomation) were used. Stained tissues were viewed by a Zeiss Axioskop 2 plus microscope with 20x magnification objective and further analyzed in L.P. Optimas 6.51 software (Media Cybernetics). Number of positive cells was estimated in ten randomly chosen view fields. All quantifications were performed in triplicates.

### Statistics, databases and patient integration

Statistical analysis was performed using Student's t-test, Friedman- test for multiple group comparisons followed by Dunns posthoc test as indicated. P-values ≤0.05 were considered statistically significant. Results are presented as means ± SD or means ± SEM as indicated. Survival analyses were performed using log rank (Mantel-Cox) tests.

## SUPPLEMENTARY MATERIALS FIGURES AND TABLES










