# Supplementary material for: Adverse genomic alterations and stemness features are induced by field cancerization in the microenvironment of hepatocellular carcinomas
Source: Oncotarget. 2017 Mar 15;8(30):48688–700. doi: 10.18632/oncotarget.16231 (PMC5564717; doi:10.18632/oncotarget.16231)
Supplement: Supplementary file 1 [file oncotarget-08-48688-s001.pdf]

Adverse genomic alterations and stemness features are induced by field cancerization in the microenvironment of hepatocellular carcinomas

Supplementary Materials

Liver metastasis

Specimens from 5 liver metastases were collected after resection at the Department of Surgery, University of Mainz, Germany following patient informed consent and local ethics committee approval. Primary tumors of the metastases included colon cancer (n=2), pancreatic cancer, sarcoma and renal cancer.

Gene expression and genomic analyses

Gene expression values were normalized by quantile normalization method across all samples following subtraction of background noises in each spot by GenomeStudio (illumina ®). Signal intensity with a detection $P > 0.05$ was treated as a missing value, and only genes with sufficient representation across the samples were included in further data analysis. Differentially expressed genes were determined using ANOVA test followed by Tukey post hoc test included in the Bioconductor package R version 3.2.2. $P < 0.005$ were considered statistically significant. Hierarchical cluster analyses were based on Pearson correlation, and complete linkage was performed with Cluster 3.0, including a filter of 80% presence for each gene. Results were visualized with TreeView 1.60 (Michael Eisen Laboratory, Lawrence Berkeley National Laboratory and University of California, Berkeley; http://rana.lbl.gov/eisen/). Ingenuity Pathway Analysis (Ingenuity Systems Inc.) and GeneGo pathways analysis (Pathway Analysis MetaCore - GeneGo Inc., St. Joseph, MI) tools were used for functional classification and network analyses. The significance of each network, function and pathway was determined by the scoring system provided by Ingenuity Pathway Analysis tool. Gene Set Enrichment analysis (GSEA) was performed using GSEA software provided by Broad Institutes (http://www.broad.mit.edu/gsea/) (1). All human gene sets from the MSigDB database were tested and gene sets with a NOM *P*-value <0.05 and FDR <0.25 were considered significantly enriched in a priori defined set of genes. For integration of patients, two different publically available microarray expression data sets were used (2,3). Genomic association analyses were performed using pLINK (version 1.07; http://pngu.mgh.harvard.edu/purcell/plink/). Only SNVs with a 90% genotyping rate and individuals with less than 10% missing genotypes were included. Adjustment for multiple testing was performed by permutation testing. The DNAcopy software was applied for CNV detection with default values. Focal somatic copy-number alterations were identified using GISTIC2.0 and adjusted according to signals from the tumor-surrounding liver (q-value<0.25) (4).

RT-qPCR

A two-step RT-qPCR, cDNA synthesis using SuperscriptIII (Invitrogen), SYBR Green Master-Mix (Bio-Rad) and *iQ5 or CFX Connect* System was performed. Oligonucleotide primers were designed using Primer3 v.0.4.0 (http://frodo.wi.mit.edu/primer3/) as described before (5). The amplification protocol was as follows: 95°C for 3 min, followed by 40 cycles of 95°C for 15 seconds and 1 minute at 60°C, completed by a dissociation curve to identify false positive amplicons. Glyceraldehyde-3-phosphate dehydrogenase (GAPDH) was used as a reference. The relative expression level of each gene was normalized to untreated cells and calculated using the formula $2^{(-\Delta\Delta Ct)}$.

REFERENCES:

1. Subramanian A, Tamayo P, Mootha VK, Mukherjee S, Ebert BL, Gillette MA, Paulovich A, Pomeroy SL, Golub TR, Lander ES, Mesirov JP. Gene set enrichment analysis: a knowledge-based approach for interpreting genome-wide expression profiles. Proc Natl Acad Sci U S A. 2005;102:15545-50.

2. Andersen JB, Factor VM, Marquardt JU, Raggi C, Lee YH, Seo D, Conner EA, Thorgeirsson SS. An integrated genomic and epigenomic

approach predicts therapeutic response to zebularine in human liver cancer. Sci Transl Med 2010;2:54ra77.

3. Roessler S, Long EL, Budhu A, Chen Y, Zhao X, Ji J, Walker R, Jia HL, Ye QH, Qin LX, Tang ZY, He P, Hunter KW,et al. Integrative genomic identification of genes on 8p associated with hepatocellular carcinoma progression and patient survival. Gastroenterology 2012;142:957-66 e12.
4. Mermel CH, Schumacher SE, Hill B, Meyerson ML, Beroukhim R, Getz G. GISTIC2.0 facilitates sensitive and confident localization of the targets of focal somatic copy-number alteration in human cancers. Genome biology 2011;12:R41.
5. Marquardt JU, Raggi C, Andersen JB, Seo D, Avital I, Geller D, Lee YH, Kitade M, Holczbauer A, Gillen MC, Conner EA, Factor VM, Thorgeirsson SS. Human hepatic cancer stem cells are characterized by common stemness traits and diverse oncogenic pathways. Hepatology 2011;54:1031-42.

Supplementary Table 1: Clinico-pathological data of HCC patients
Supplementary Table 2: List of antibodies
Supplementary Table 3: Gene expression signatures for the different regions
Supplementary Table 4: The top 5 functional networks for each

For Supplementary Tables 1-4 see in Supplementary Files.

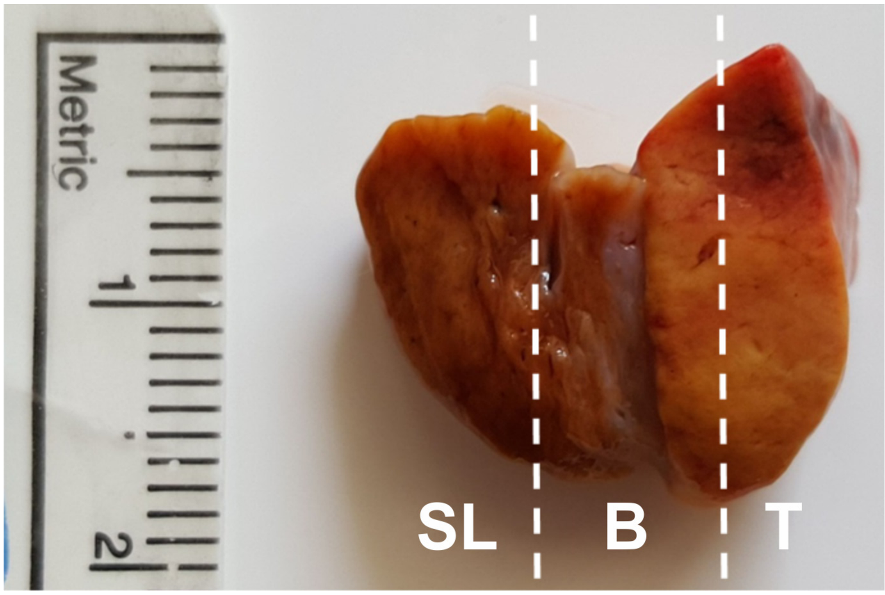

Supplementary Figure 1: Macroscopic image of the different regions Image displaying how the different regions were macroscopically dissected. Dashed lines showing the approximate cutting point for the border region (approximately 2-4 mm on each side).

A)

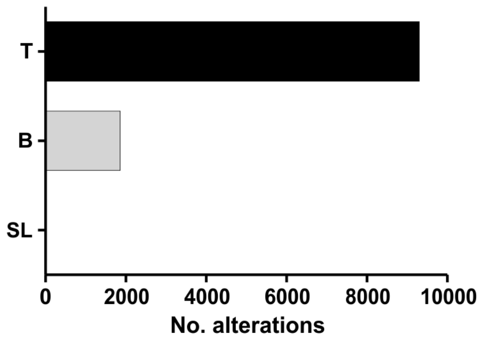

B)

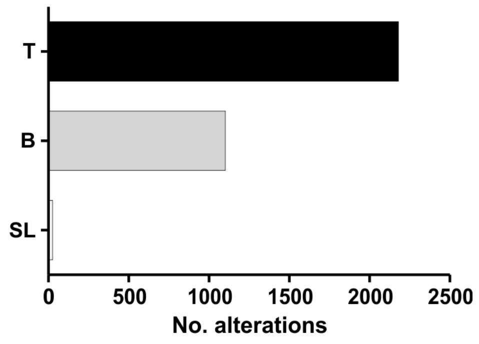

C)

Chrom.	SL	B	T
1		798	1785
2	1	18	680
3	3	31	180
4		13	122
5	1	12	1058
6		62	693
7	1	126	1482
8	3	367	1196
9	1	47	95
10	3	236	397
11		3	103
12	3	9	29
13			101
14			310
15		46	174
16			150
17	1	4	363
18			2
19	1	21	104
20		21	178
21		3	34
22	5	40	65

D)

Chrom.	SL	B	T
1	4	76	27
2	1	86	13
3		69	26
4	3	84	212
5		53	29
6	3	152	432
7	4	54	42
8	5	97	261
9	1	65	92
10	2	59	102
11		47	27
12		51	44
13	1	48	183
14		30	124
15		17	6
16		10	90
17		18	168
18	1	28	6
19		6	28
20		7	1
21		43	85
22	1	2	182

Supplementary Figure 2: Number of genetic alteration of the different regions. Number of genetic alterations for each region determined by pennCNV. A) shows total genetic gains; B) corresponding total number of losses (C-D) Genetic variation (gains (C) and losses (D) for each chromosome.

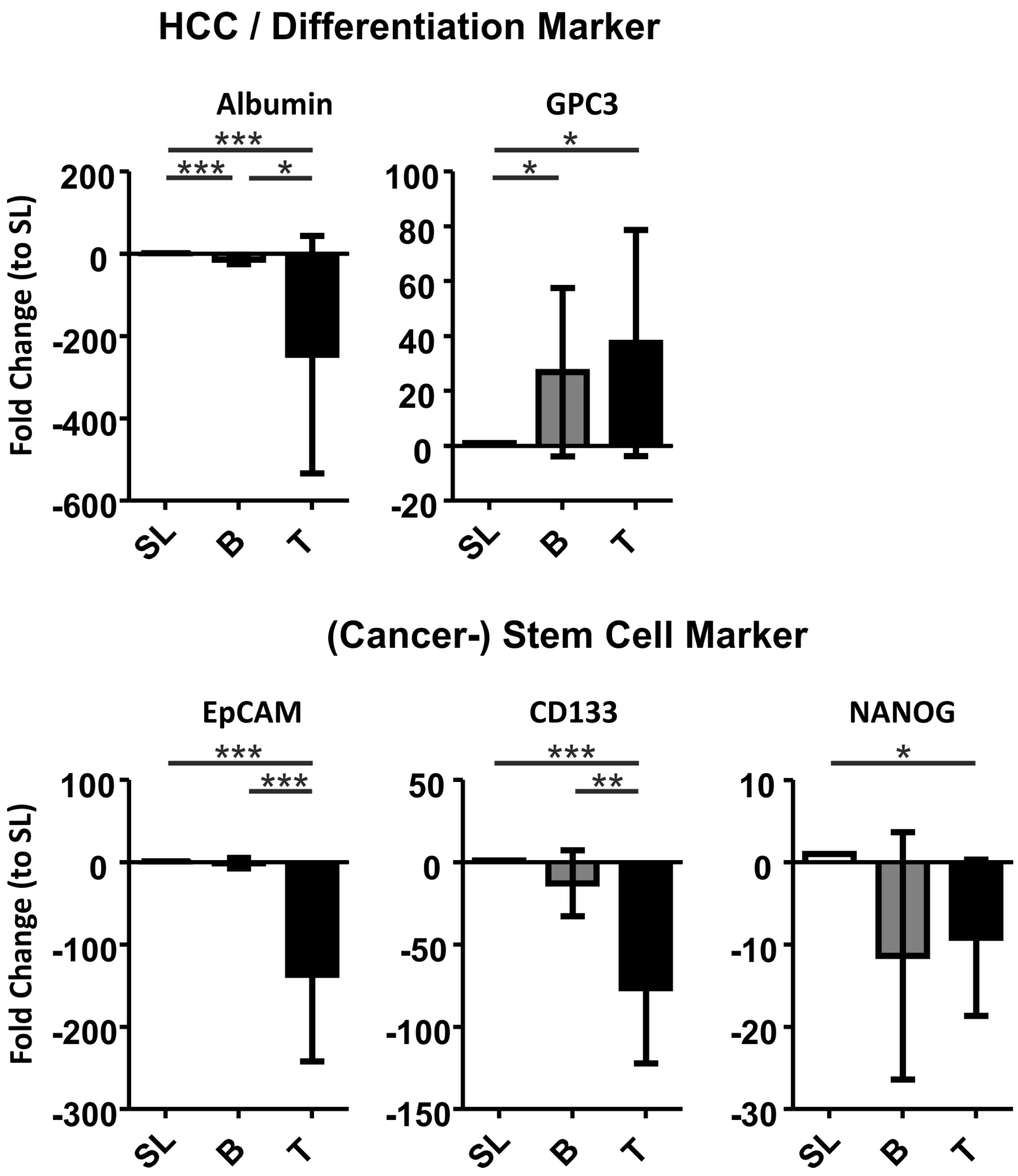

Supplementary Figure 3: Activation of selected markers by qRT-PCR. qRT-PCR results for selected HCC (GPC3) and differentiation (Albumin) (upper panels) as well as (cancer-) stem cell markers (lower panels). The gene expression was analysed by Friedman- test for multiple group comparisons followed by Dunns posthoc test. (n=28; P-values: *≤ 0.05; **≤ 0.05; ***≤ 0.001). The data are presented as mean fold differences ± SD using 2^-ddCT normalized to surrounding liver. GAPDH was used as internal control.

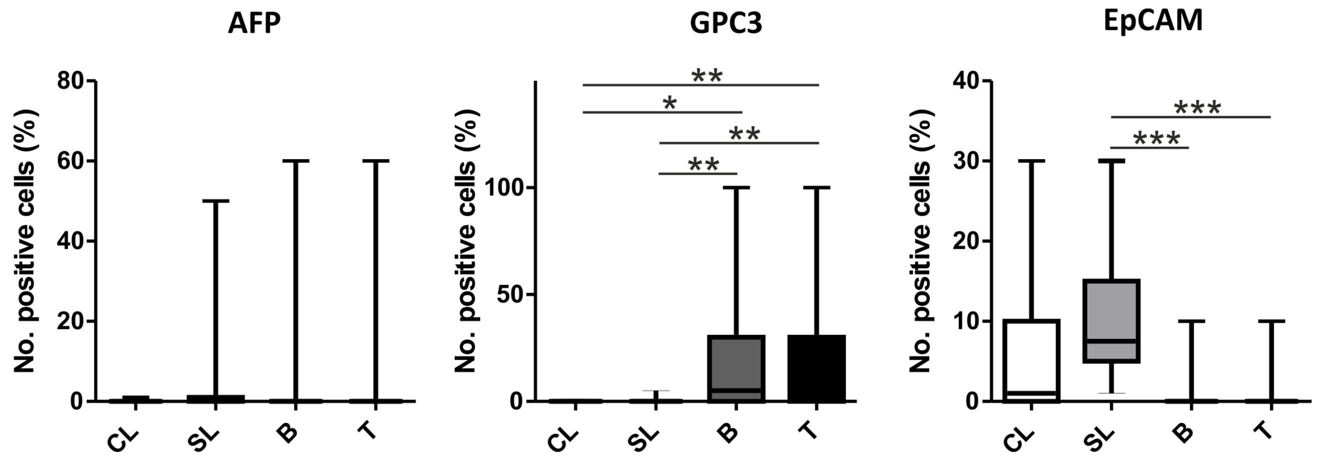

Supplementary Figure 4. Validation of selected markers in independent patient cohorts. Expression of selected markers was validated by immunohistochemistry. Estimation of the number of positive cells based on 10 randomly selected view fields is displayed. (T=tumor, B=border, SL=surrounding liver; CL=(non-tumorous) cirrhotic liver) Statistical evaluation based on Friedman- test for multiple group comparisons followed by Dunns posthoc test. (n=20, CL=10; P-values: *≤ 0.05; **≤ 0.05; ***≤ 0.001)

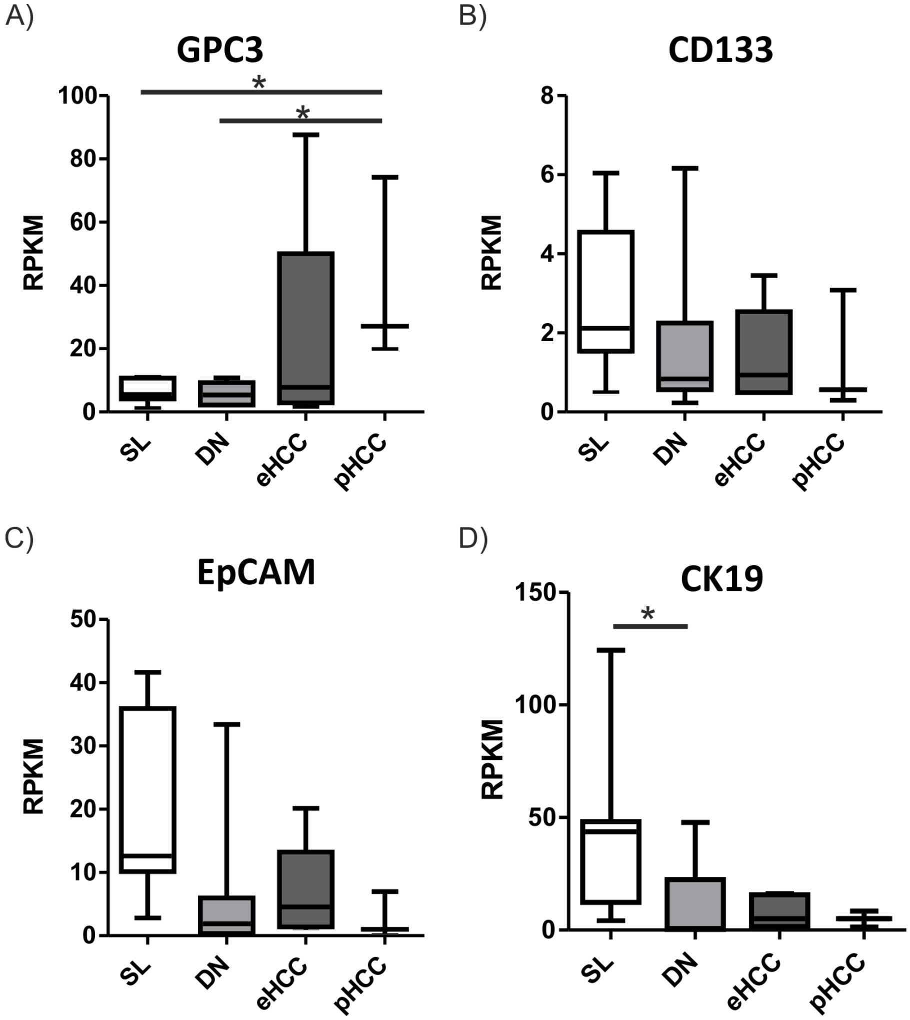

Supplementary Figure 5. Activation of selected markers in different pre-neoplastic and cancerous lesions by RNA sequencing. Expression of selected HCC (GPC3) (A) and (cancer-) stem cell markers (B-D) using previously published dataset of different lesion representing the whole spectrum of lesions in hepatocarcinogenesis (SL= surrounding liver; DN=dysplastic lesions; eHCC= early HCC; pHCC= progressed HCC). Graphical representation of RPKM and quantile normalized reads. (n=28; P-values: *≤ 0.05; **≤ 0.05; ***≤ 0.001). The data are presented as mean fold differences ± SD.

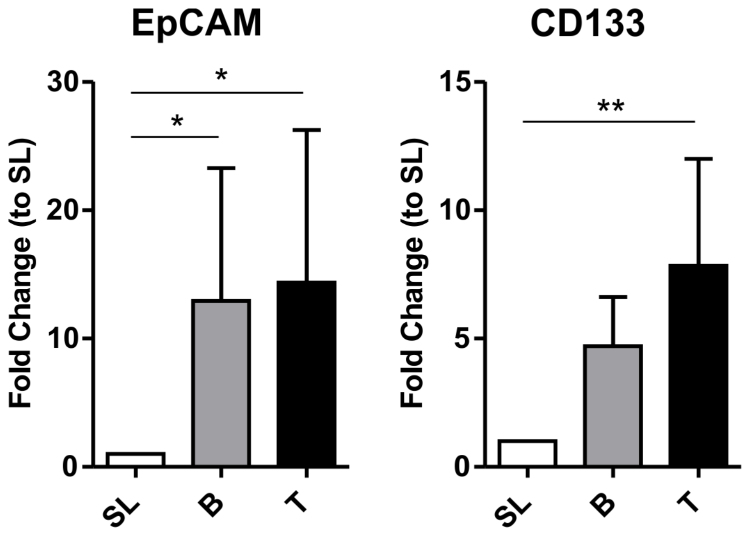

Supplementary Figure 6: Activation of selected markers in liver metastasis by qRT-PCR. qRT-PCR results for selected (cancer-) stem cell markers. The data are presented as mean fold differences ± SD using 2^-ddCT normalized to surrounding liver. GAPDH was used as internal control (M=metastasis, B=border, SL=surrounding liver).

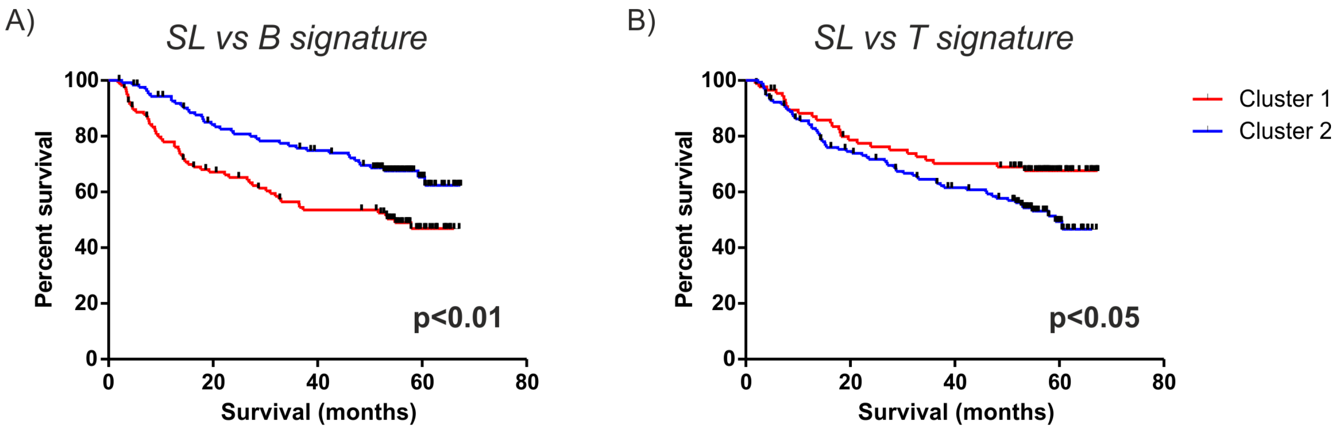

Supplementary Figure 7: Validation of the prognostic implications of the SL vs B and B vs T signature in an independent cohort of HCC patients. Integration of the 590 gene SL vs B (A) and 100 gene SL vs T (B) signature in a previously published dataset of 247 HCC patients of which survival data was available for 241 patients (3). Shown are the Kaplan-Meier plots of overall survival.
