# Supplementary material for: Adverse genomic alterations and stemness features are induced by field cancerization in the microenvironment of hepatocellular carcinomas
Source: Oncotarget. 2017 Mar 15;8(30):48688–700. doi: 10.18632/oncotarget.16231 (PMC5564717; doi:10.18632/oncotarget.16231)
Supplement: Supplementary file 2 [file oncotarget-08-48688-s002.docx]

Table 1A. Mainz cohort

|  | **No. Pat. (n=28)** | **%** |
| --- | --- | --- |
| m | 21 | 75 |
| w | 7 | 25 |
| HBV | 3 | 11 |
| HCV | 10 | 36 |
| Alcohol | 8 | 28 |
| NASH | 3 | 11 |
| Unkown | 4 | 14 |
| BCLC A | 12 | 43 |
| BCLC B | 12 | 43 |
| BCLC C | 4 | 14 |
| Cirrhosis | 17 | 61 |
| G1 | 5 | 18 |
| G2 | 6 | 21 |
| G3 | 17 | 61 |
| Size >5cm | 17 | 61 |
| Vasc. Invasion | 7 | 25 |

Table 1B. Swiss cohort

|  | **No. Pat. (n=20)** | **%** |
| --- | --- | --- |
| m | 16 | 80 |
| w | 4 | 20 |
| HBV | 1 | 5 |
| HCV | 6 | 30 |
| Alcohol | 8 | 40 |
| NASH | 1 | 5 |
| Unkown | 4 | 20 |
| BCLC A | 15 | 75 |
| BCLC B | 2 | 10 |
| BCLC C | 1 | 5 |
| unknown | 2 | 10 |
| G1 | 0 | 0 |
| G2 | 8 | 40 |
| G3 | 12 | 60 |
| Size >5cm | 8 | 40 |
| Vasc. Invasion | 2 | 10 |
