# Supplementary material for: Adverse genomic alterations and stemness features are induced by field cancerization in the microenvironment of hepatocellular carcinomas
Source: Oncotarget. 2017 Mar 15;8(30):48688–700. doi: 10.18632/oncotarget.16231 (PMC5564717; doi:10.18632/oncotarget.16231)
Supplement: Supplementary file 3 [file oncotarget-08-48688-s003.docx]

| \| **Antigen** \| **Dilution** \| **Manufacturer** \| \| --- \| --- \| --- \| \| AFP \| 1:400 \| Dako \| \| CD133 \| 1:20 \| Miltenyi Biotech \| \| EpCAM \| 1:80 \| Cell Signaling \| \| CD3 \| 1:100 \| Santa Cruz Biotechnology \| \| CD68 \| 1:70 \| Dako \| \| Ki67 \| 1:200 \| Rockland \| \| Anti-mouse IgG Alexa Fluor 488 \| 1:500 \| Cell Signaling \| \| Anti-rabbit IgG Alexa Fluor 555 \| 1:500 \| Cell Signaling \| \|  \|  \|  \| \|  \|  \|  \| \|  \|  \|  \| \|  \|  \|  \| \|  \|  \|  \| |  |
| --- | --- | --- | --- | --- | --- | --- | --- | --- | --- | --- | --- | --- | --- | --- | --- | --- | --- | --- | --- | --- | --- | --- | --- | --- | --- | --- | --- | --- | --- | --- | --- | --- | --- | --- | --- | --- | --- | --- | --- | --- | --- | --- | --- |
|  |  |
|  |  |
|  |  |
|  |  |
|  |  |
|  |  |
