# Supplementary material for: Adverse genomic alterations and stemness features are induced by field cancerization in the microenvironment of hepatocellular carcinomas
Source: Oncotarget. 2017 Mar 15;8(30):48688–700. doi: 10.18632/oncotarget.16231 (PMC5564717; doi:10.18632/oncotarget.16231)
Supplement: Supplementary file 5 [file oncotarget-08-48688-s005.docx]

***Surrounding liver vs tumor signature***

| **#** | **Networks** | **p-Value** | **FDR** | **Ratio** |
| --- | --- | --- | --- | --- |
| 1 | Transcription_mRNA processing | 8.57E-12 | 1.36E-09 | 56/160 |
| 2 | Translation_Translation initiation | 3.93E-08 | 3.13E-06 | 51/171 |
| 3 | Cell cycle_Mitosis | 1.99E-07 | 1.06E-05 | 51/179 |
| 4 | Proteolysis_Ubiquitin-proteasomal proteolysis | 7.34E-07 | 2.92E-05 | 47/166 |
| 5 | Transcription_Chromatin modification | 2.43E-06 | 7.73E-05 | 38/128 |

***Surrounding liver vs border signature***

| **#** | **Networks** | **p-Value** | **FDR** | **Ratio** |
| --- | --- | --- | --- | --- |
| 1 | Transcription_Chromatin modification | 2.36E-04 | 1.66E-02 | 13/128 |
| 2 | Inflammation_IL-6 signaling | 4.37E-04 | 1.66E-02 | 12/119 |
| 3 | Immune response_Antigen presentation | 6.11E-04 | 1.86E-02 | 16/197 |
| 4 | Proteolysis_Ubiquitin-proteasomal proteolysis | 9.23E-04 | 2.34E-02 | 14/166 |
| 5 | Cell cycle_G1-S Growth factor regulation | 1.04E-02 | 1.49E-01 | 13/195 |

***Border vs tumor signature***

| **#** | **Networks** | **p-Value** | **FDR** | **Ratio** |
| --- | --- | --- | --- | --- |
| 1 | Inflammation_IL-6 signaling | 7.62E-04 | 5.18E-02 | 5/119 |
| 2 | Inflammation_Inflammasome | 5.77E-03 | 1.96E-01 | 4/118 |
| 3 | Transcription_Transcription by RNA polymerase II | 1.61E-02 | 3.64E-01 | 4/159 |
| 4 | Development_Regulation of angiogenesis | 4.74E-02 | 6.83E-01 | 4/223 |
| 5 | DNA damage_MMR repair | 5.09E-02 | 6.83E-01 | 2/59 |
